# Factor Structure, Validity, and Reliability of the STarT Back Screening Tool in Italian Obese and Non-obese Patients With Low Back Pain

**DOI:** 10.3389/fpsyg.2021.740851

**Published:** 2021-10-20

**Authors:** Emanuele Maria Giusti, Giorgia Varallo, Alessandra Abenavoli, Gian Mauro Manzoni, Luca Aletti, Paolo Capodaglio, Gianluca Castelnuovo, Alberto Maggiani

**Affiliations:** ^1^Department of Psychology, Catholic University of Milan, Milan, Italy; ^2^Psychology Research Laboratory, Ospedale San Giuseppe, Istituto Auxologico Italiano IRCCS, Verbania, Italy; ^3^Research Department, Accademia Italiana di Medicina Osteopatica (AIMO), Saronno, Italy; ^4^Faculty of Psychology, eCampus University, Novedrate, Italy; ^5^Rehabilitation Unit and Research Laboratory in Biomechanics and Rehabilitation, Istituto Auxologico Italiano IRCCS, San Giuseppe Hospital, Verbania, Italy; ^6^Department Surgical Sciences, Physical Medicine and Rehabilitation, University of Turin, Turin, Italy

**Keywords:** back pain, obesity, prognosis, risk factors, rehabilitation, STarT back screening tool, validity, reliability

## Abstract

**Background:** The STarT Back Screening Tool (SBST) is a self-report questionnaire developed for prognostic purposes which evaluates risk factors for disability outcomes in patients with chronic low back pain. Previous studies found that its use enables to provide a cost-effective stratified care. However, its dimensionality has been assessed only using exploratory approaches, and reports on its psychometric properties are conflicting.

**Objective:** The objective of this study was to assess the factorial structure and the psychometric properties of the Italian version of the STarT Back Screening Tool (SBST).

**Materials and Methods:** Patients with medical diagnosis of low back pain were enrolled from a rehabilitation unit of a tertiary care hospital specialized in obesity care (Sample 1) and from a clinical internship center of an osteopathic training institute (Sample 2). At baseline and after 7 days patients were asked to fill a battery of self-report questionnaires. The factorial structure, internal consistency, test-retest reliability, and construct validity of the SBST were assessed.

**Results:** One hundred forty-six patients were enrolled (62 from Sample 1 and 84 from Sample 2). The confirmatory factor analysis showed that the fit of the original two-correlated factors model was adequate (CFI = 0.98, TLI = 0.99, RMSEA = 0.03). Cronbach's α of the total scale (α = 0.64) and of the subscales (physical subscale α = 0.55; psychological subscale α = 0.61) was below the cutoffs, partly because of the low correlation of item 2 with the other items. Test-retest reliability was adequate (ICC = 0.84). The SBST had moderate correlations with comparisons questionnaires, except for the Roland-Morris Disability Questionnaire, which had a high correlation (*r* = 0.65).

**Discussion:** The SBST has adequate psychometric properties and can be used to assess prognostic factors for disability in low back pain patients.

## Introduction

Low back pain is a very common symptom with a mean point prevalence of 18.3%, a 1-month prevalence of 30.8% and a lifetime prevalence between 70 and 80%, with about 58% of the sufferers seeking medical care (Ferreira et al., 2010; Maher et al., 2017). Low back pain has a highly disabling potential due to its impact on mobility, sleep, performing activities of daily living, independence, and participation to activities (De Souza and Frank, 2007). These limitations are often accompanied by a strong psychological burden, consisting of feelings of anxiety, depression, catastrophizing, and difficulties in coping with pain (Castelnuovo et al., 2016). These aspects, in turn, worsen the perception of disability (Ranger et al., 2020). The threat posed by this vicious circle is even greater when obesity is comorbid. Obesity is a risk factor for low back pain due to underlying biological processes and the two conditions share risk factors including age, sex, race/ethnicity, and reduced physical activity (Heuch et al., 1976; Shiri et al., 2010). Since in 2–7% of the cases low back pain becomes chronic and causes long-lasting disability, it is paramount to identify those at risk for poor treatment outcomes when they are referred to therapeutic programs (da C Menezes Costa et al., 2012; James et al., 2018).

Rehabilitation in patients with low back pain, with or without comorbid obesity, includes pharmacological treatment, exercise, manual therapy, and psychological treatments (Shipton, 2018; Giusti et al., 2020; National Institute for Health Care Excellence, 2020). These treatments have shown to be effective in reducing pain and disability, especially if they are provided in multidisciplinary settings (Kamper et al., 2015). Nonetheless, between 30 and 40% of patients do not respond to treatment (Cecchi et al., 2012). Variables predicting treatment and disability outcomes include demographic, e.g., age, physical, e.g., duration of the pain episode, pain intensity and pain-related disability, and psychological variables, e.g., catastrophizing, anxiety, depression, kinesiophobia (Hayden et al., 2009; George and Beneciuk, 2015). Since a percentage of patients referred to rehabilitation treatment, it is necessary to have instruments assessing these risk factors that can be employed to quickly identify them.

The STarT Back Screening Tool (SBST) was developed for this purpose (Hill et al., 2008). The SBST is a brief self-report questionnaire. consisting in nine items with a binary response system (i.e., “agree” or “disagree”) investigating known physical (i.e., referred leg pain, comorbid pain, disability related to walking, disability related to dressing) and psychological (i.e., catastrophizing, fear, anxiety, depression, and bothersomeness) modifiable risk factors for worse disability outcomes. Based on a biopsychosocial approach, the SBST is intended as a component of a stratified model of care management of low back pain. In this model, the patient is allocated into one of three risk-based groups (low, medium, and high risk) based on his/her SBST score. Then, the patient receives a treatment whose components are in proportion to the risk (e.g., patients in the high risk groupphysical therapy, psychological treatments) varies according to the risk (Hill et al., 2011). The scoring system proposed by the authors is as follows. Patients with total scores between 0 and 3 are considered at low risk, patients with total scores ≥ 4 but with scores <4 in the psychological subscale are considered at medium risk and patients with total scores ≥ 4 and scores ≥4 in the psychological subscale at high risk (Hill et al., 2008; Shiri et al., 2010). Research using this scoring system or the SBST total scores found that these scores predict 3- and 6-months disability (Hill et al., 2008; Ami et al., 2020), health-related quality of life, work ability (Forsbrand et al., 2018) and functional recovery after physical treatments (Katzan et al., 2019). Furthermore, implementation of stratified care based on scores of the SBST was clinically and cost-effective, also in the long term, compared to usual non-stratified care (Hill et al., 2011; Whitehurst et al., 2012; Hall et al., 2020).

Research on the psychometric properties of the SBST shows that the questionnaire and its translated versions have good or excellent test-retest reliability (Luan et al., 2014; Raimundo et al., 2017; Robinson and Dagfinrud, 2017; Yilmaz Yelvar et al., 2019; Ami et al., 2020; Schmidt and Naidoo, 2020), construct validity (Hill et al., 2008; Luan et al., 2014; Yilmaz Yelvar et al., 2019; Ami et al., 2020) and responsiveness (Wideman et al., 2012). Estimates of the internal consistency of this questionnaire are more heterogeneous, with some studies showing good internal consistency (Hill et al., 2008; Raimundo et al., 2017; Yilmaz Yelvar et al., 2019; Schmidt and Naidoo, 2020) and other studies showing inadequate internal consistency (Karstens et al., 2015; Piironen et al., 2016; Robinson and Dagfinrud, 2017). None of these studies, however, also tested the psychometric properties of the SBST in patients with low back pain and comorbid obesity. Moreover, the factorial structure of the questionnaire has been rarely addressed. To our knowledge, the distinction between the physical and psychological items, the absence of other sources of variability, and the presence of a correlation between the latent factors have not been formally checked with confirmatory approaches. Knowledge about these properties is needed to justify the calculation of the subscale scores and of the total score.

The Italian translation of the SBST has been performed and has proven to be linguistically accurate, easy to understand, and acceptable for use by Italian-speaking patients (Maggiani and Abenavoli, 2019). However, its reliability and construct validity has not been addressed yet. The validation of the Italian version of the SBST in both obese and non-obese patients with low back pain could be useful to help determine the appropriate prognosis and treatment pathways. Therefore, the objective of this study was to assess the factorial structure of the Italian version of the SBST and to evaluate its internal consistency, test-retest reliability, and construct validity.

## Materials and Methods

To perform this study, data from two samples were used. Sample 1 included consecutive obese patients admitted to the Rehabilitation Unit and Research Laboratory in Biomechanics and Rehabilitation of the San Giuseppe Hospital, Istituto Auxologico Italiano, during the first week of a 4-week comprehensive rehabilitation program and weight loss management, and being referred for medical attention for low back pain. Sample 2 included consecutive patients referring to the training and clinical internship center of the Italian Academy of Osteopathic Medicine AIMO, Saronno, Italy. In both samples, inclusion criteria were having medical diagnosis of low back pain not explained by trauma or other evident conditions and age between 18 and 80 years. The diagnosis was confirmed by a physical and rehabilitation physician at admission.

Low back pain was defined as pain or discomfort between the costal margins and superior gluteal line, with or without leg pain. Patients were excluded if they were not able to provide informed consent.

After being enrolled, participants were asked to fill a short battery of self-report questionnaires including the SBST. After 7 days, in the occasion of a subsequent visit, they were asked to fill the SBST.

All procedures performed in studies involving human participants were in accordance with the ethical standards of the institutional and/or national research committee and with the 1964 Helsinki declaration and its later amendments or comparable ethical standards. Informed consent was obtained from all participants included in the study prior to accessing the questionnaires. This study was approved by the local institutional review board (code I2019/01001).

### Measurement Instruments

Along with the SBST, the battery of self-report questionnaires administered at the baseline included:

- A 11-points pain Numeric Rating Scale (NRS) assessing the intensity of current pain from 0 (“No pain”) to 10 (“Worst possible pain”).
- The Roland-Morris Disability Questionnaire (RMDQ) (Padua et al., 2002). The RMDQ is a valid and reliable measure of pain-related disability consisting of 24 items listing limitations to common daily activities rated on a binary response system (“yes” or “no”). Higher values indicate a higher disability. In this study, we employed the Italian version of the RMDQ, which was cross-culturally adapted for the use with Italian patients and which showed good test-retest reliability and internal consistency (Padua et al., 2002).
- The Pain Catastrophizing Scale (PCS) (Monticone et al., 2012). The PCS is a self-report scale measuring pain catastrophizing using 13 items on a 5-point Likert-type scale ranging from 0 (“not at all”) to 4 (“all the time”). The PCS assesses thoughts and feelings associated with pain experience. In this study, we employed the total score, with higher values indicating higher levels of catastrophizing. The Italian version of PCS has received extensive validation and showed good internal consistency, test-retest reliability, and concurrent validity (Monticone et al., 2012).
- The Tampa Scale of Kinesiophobia (TSK) (Monticone et al., 2010). The TSK is a 17-item questionnaire using a 4-point Likert scale ranging from 1 (“completely disagree”) to 4 (“completely agree”) that was developed as a measure of pain-related fear of movement. Higher values indicate a higher fear of movement. The TSK was cross-culturally adapted for the use with Italian patients and the Italian version showed good structural validity, internal consistency, test-retest reliability, and discriminant validity (Monticone et al., 2010).
- The European Quality of Life Instrument (EQ-5D) (Scalone et al., 2013). The EQ-5D is a self-report questionnaire which includes five items assessing mobility, self-care, usual activities, pain/discomfort, and anxiety/depression. The Italian version of the EQ-5D has been validated and normative values are available (Savoia et al., 2006; Scalone et al., 2013).

The battery administered after 7 days from baseline included the SBST, a pain NRS and a single question on a 7-point scale ranging from 0 (“No improvement”) to 6 (“Complete recovery”) measuring perception of improvement from the baseline.

### Statistical Analysis

Frequency and percentages were used to examine categorical variables. Medians and Interquartile Ranges (IQR) were used to describe ordinal variables, whereas means and standard deviations were used to describe interval or ratio variables. Differences between the two samples regarding demographic and clinical variables were assessed using chi-square, Mann-Whitney and *t*-tests, as appropriate. Furthermore, sex differences regarding the SBST scores were assessed using Mann-Whitney tests. The amount of missing data was <5% and were therefore excluded from the analyses.

To assess the structural validity of the SBST, we performed a confirmatory factor analysis evaluating a two correlated factors model distinguishing a physical (items 1–4) and a psychosocial (items 5–9) subscale (Abedi et al., 2015). Parameters estimation was performed using a diagonally weighted least squares estimator with robust standard errors. The fit of the model was considered adequate if the Root Mean Square Error of Approximation (RMSEA) was < 0.06, the Tucker Lewis Index (TLI) and the Comparative Fit Index (CFI) were > 0.95. To evaluate the contribution of each item to the respective subscale, item loadings were examined. Item loadings were considered excellent if ≥0.71, very good if <0.71 and ≥0.63, good if <0.63 and ≥0.55, fair if <0.55 and ≥0.45, poor if <0.45 and ≥0.32 and very poor if <0.32 (Tabachnick et al., 2007).

To assess the internal consistency of the SBST and of its subscales, the Cronbach's α was computed. The cut-off for adequate internal consistency was 0.70 (Prinsen et al., 2018). In addition, the “α if item deleted” technique was used to identify whether an item's deletion enhanced the Cronbach's α coefficient. Then, to estimate test-retest reliability, the Intraclass Correlation Coefficient (ICC) was used using the SBST scores at the baseline and at 7 days. Patients who reported during the second administration that their pain had sufficiently, mostly or completely resolved at the single question investigating their perception of improvement were excluded from this analysis. To calculate the ICC, a two-way mixed-effect ANOVA model with interaction for the absolute agreement between single scores was used (ICC_3,k_) (Qin et al., 2019). Values ≤0.5 indicate poor reliability, values >0.5 and ≤0.75 indicate moderate reliability, values >0.75 and ≤0.9 indicate good reliability, and values >0.90 indicate excellent reliability.

To assess construct validity, a set of pre-specified hypotheses regarding the correlations between the SBST and the comparisons questionnaires was formulated (Reeve et al., 2013). Associations were inspected using Pearson's r. We expected that the total score of the SBST should have moderate correlation (*r* >0.3 and <0.6) with the NRS, PCS, TSK, RMDQ, and EQ-5D scales.

The threshold for the identification of significant values was α = 0.05. The analyses were performed using the R (version 3.6.0) packages *lavaan* (confirmatory factor analysis), *psych* (internal consistency and test-retest reliability), and *base* (correlations).

## Results

### Description of the Sample

Sixty-two patients were enrolled in Sample 1 and 84 in Sample 2, for a total amount of 146 patients. Demographic and clinical characteristics of the samples are reported in Table 1. Patients from Sample 1 were older and had higher catastrophizing, disability, and kinesiophobia scores, as well as higher baseline SBST scores than patients from Sample 2. No sex differences regarding the SBST scores were detected (total scale: *U* = 2,758, *p* = 0.71; psychological subscale: *U* = 2825.5, *p* = 0.51; physical subscale: *U* = 2628.5, *p* = 0.89).

**Table 1:** Frequencies and descriptive statistics of the sample.

		**Total sample**	**Sample 1**	**Sample 2**	
		**(*n* = 146)**	**(*n* = 62)**	**(*n* = 84)**	***p*-value^a^**
Age		55.4 (13.2)	59.1 (8.9)	52.6 (15.2)	<0.01
Sex	Male	52 (35.9)	23 (37.1)	29 (34.9)	
	Female	93 (64.1)	39 (62.9)	54 (65.1)	0.92^b^
PCS		17 (10.1)	21 (11)	14 (8.3)	<0.01
EQ5D		0.7 (0.2)	0.7 (0.2)	0.7 (0.1)	0.47
RMDQ		8.5 (5.9)	11.4 (6.2)	6.3 (4.7)	<0.01
TSK		27.4 (7.4)	29.5 (7.4)	25.9 (7)	<0.01
NRS t0		5.8 (2.2)	6.2 (2.4)	5.5 (2.1)	0.06
NRS t1		3.8 (2.4)	3.2 (2.5)	4.2 (2.1)	0.01
Perception of improvement		5 [4, 6]	3 [2, 5]	4 [3, 6]	<0.01^c^
SBST-Ph t0		2.1 (1.2)	2.6 (1.2)	1.8 (1.1)	<0.01
SBST-Ps t0		1.1 (1.3)	1.2 (1.5)	0.9 (1)	0.15
SBST total t0		3.2 (2)	3.9 (2.2)	2.7 (1.8)	<0.01
SBST-Ph t1		1.5 (1.2)	1.3 (1.3)	1.6 (1.1)	0.10
SBST-Ps t1		0.7 (1.1)	0.6 (1.1)	0.8 (1.1)	0.28
SBST total t1		2.2 (1.9)	1.9 (2.1)	2.4 (1.8)	0.11

*Frequencies and percentages are reported for categorical variables, medians and interquartile ranges for ordinal variables and means and standard deviations for interval or ratio variables*.

a *p-values are based on independent sample t-test, if not otherwise specified*.

b *based on Chi square test*.

c *based on Mann-Whitney test. PCS, Pain Catastrophizing Scale; RMDQ, Roland Morris Disability Questionnaire; TSK, Tampa Scale for Kinesiophobia; NRS, Numeric Rating Scale; SBST-Ph, Start Back Screening Tool—Physical subscale; SBST-Ps, Start Back Screening Tool—Psychological subscale*.

### Confirmatory Factor Analysis

**Figure 1:**
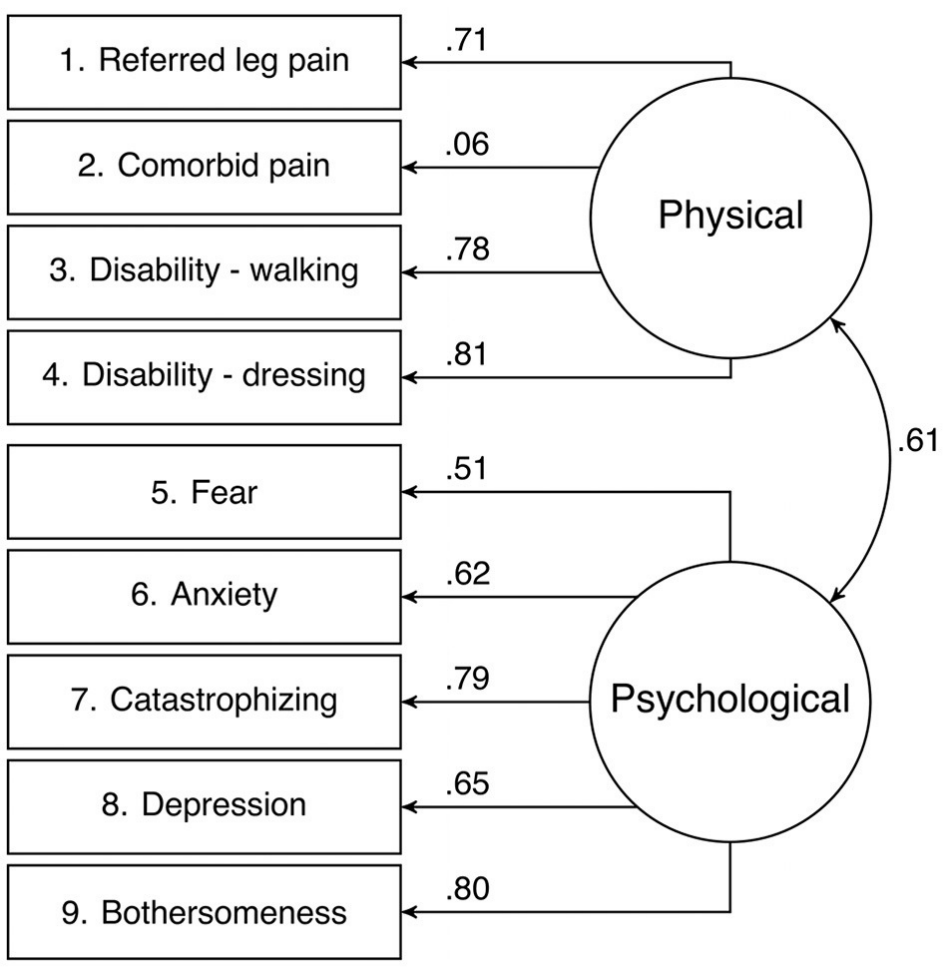
Confirmatory factor analysis of the Start Back Screening Tool evaluating a two correlated factors model.

The fit of the two correlated factors model was adequate: CFI = 0.98, TLI = 0.99, RMSEA = 0.03. Items 1, 3, and 4 had excellent loadings on the physical subscale, whereas item 2 had a very poor loading. Regarding the psychological subscale, item 5 had a fair loading, item 6 had a good loading, item 8 had a very good loading and item 7 and 9 had excellent loadings (Figure 1). The physical and psychological subscale had a correlation of 0.61. Overall, the two correlated factors model was considered adequate and subscales scores were used in addition to the total score in the reliability and validity analyses.

### Reliability

The Cronbach's α of the total scale was 0.64 in the total sample. The Cronbach's α in Sample 1 was 0.68 and in sample 2 was 0.55. The Cronbach's α of the physical subscale was 0.55 (0.58 in sample 1 and 0.53 in sample 2), whereas the Cronbach's α of the psychological subscale was 0.61 (0.73 in sample 1 and 0.55 in sample 2). The α if item deleted revealed that removing item 2 would increase the internal consistency of the total scale to 0.70 and the one of the physical subscale to 0.65.

The ICC of the total scale was 0.84 in the total sample, indicating good test-retest reliability. The ICC in Sample 1 and 2 were 0.85 and 0.84, respectively. Regarding the physical subscale, the ICC was 0.77 in the total sample, indicating good test-retest reliability, and 0.75 and 0.84 in Sample 1 and 2, respectively. Finally, the ICC of the psychological subscale was 0.84 in the total sample and 0.85 and 0.84 in Sample 1 and 2, respectively. These values indicate good test-retest reliability.

### Construct Validity

**Figure 2:**
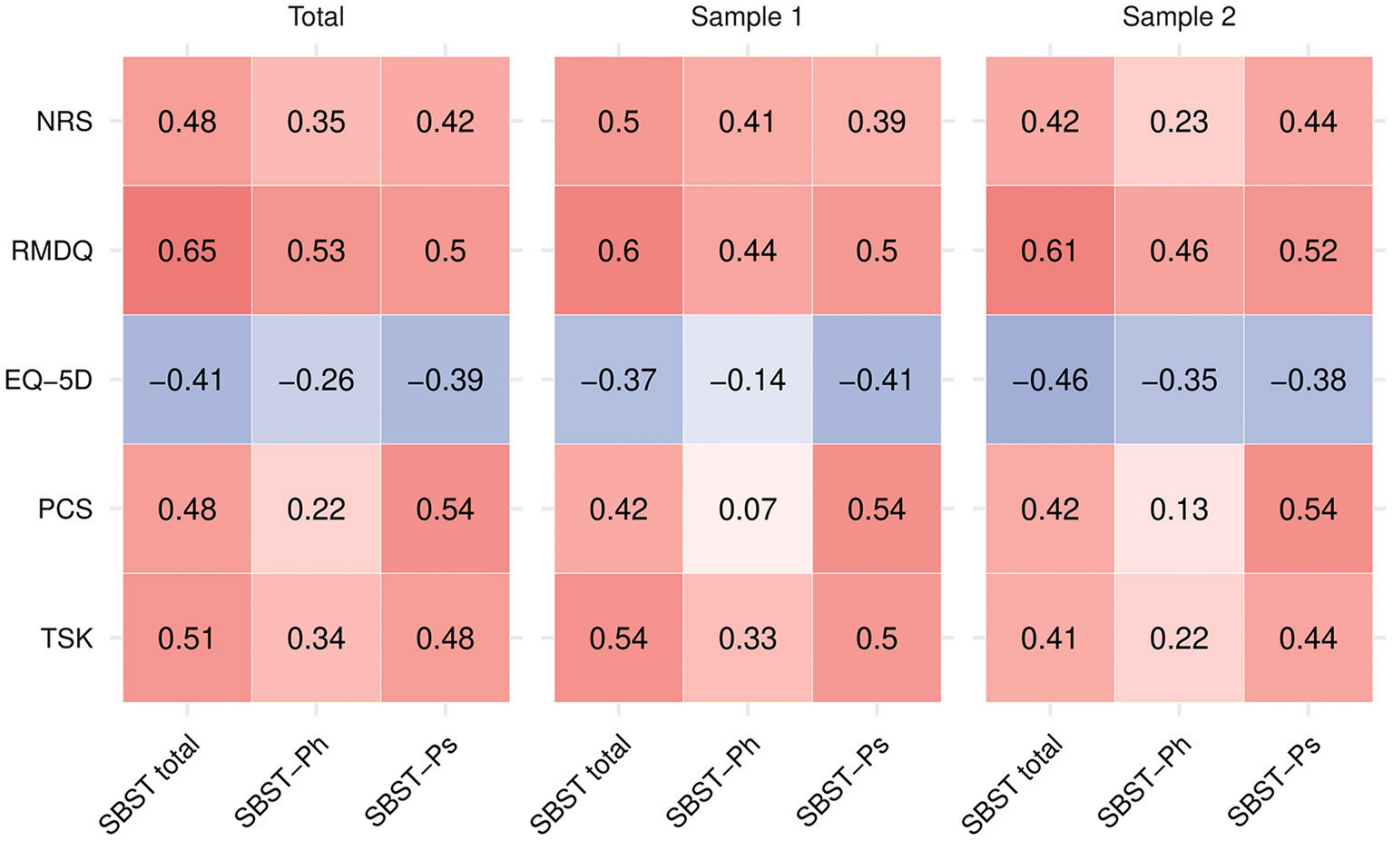
Correlations between the Start Back Screening Tool and the comparator instruments. PCS, Pain Catastrophizing Scale; RMDQ, Roland Morris Disability Questionnaire; TSK, Tampa Scale for Kinesiophobia; NRS, Numeric Rating Scale; SBST-Ph, Start Back Screening Tool—Physical subscale; SBST-Ps, Start Back Screening Tool—Psychological subscale.

The correlations between the SBST total scale and subscales in the total sample and in Sample 1 and 2 are reported in Figure 2. Most of the hypotheses were met in all samples. The only hypotheses that were not met regarded the correlations between the SBST total scale and the RMDQ in the total sample and in Sample 2, which were slightly higher than 0.60.

## Discussion

The aim of the present study was to assess the factorial structure, test-retest reliability and construct validity of the SBST in a sample of Italian patients with low back pain with or without comorbid obesity. The results suggest that this instrument assesses the physical and psychological characteristics of the patient's pain experience, that it has adequate internal consistency and good test-retest reliability and construct validity.

To our knowledge, this study is the first report presenting a confirmatory factor analysis of the SBST. Similarly to our study, but using an exploratory procedure, Abedi et al. (2015) found that a two-factor model had a good fit with their data. The presence of two factors is consistent with the categorization of the items made by the authors of the original questionnaire, who divided the items based on their physical or psychological content (Hill et al., 2008). The presence of a correlated-factor structure suggests that subscale scores can be calculated, and the presence of a moderate correlation between the two subscales provides a rationale for the use of the total score. However, to provide a more sound argument for the use of the total score, future studies should assess if the SBST is “unidimensional enough” to allow for its calculation (Reise et al., 2013).

Similarly to other studies on translated versions of the SBST, the internal consistency of the total scale and of the subscales was below the cutoff (Karstens et al., 2015; Piironen et al., 2016; Robinson and Dagfinrud, 2017). Part of this lack of internal consistency was due to the poor performance of item 2, since it had a low correlation with the physical factor in the confirmatory factor analysis and its removal would improve the internal consistency of the total scale and of the physiological scale, as found in another study (Robinson and Dagfinrud, 2017). This could be due to the fact that this item assesses comorbid neck or shoulder pain, which could be uncorrelated with other physical characteristics of the patient's pain experience. In our opinion, the poor internal consistency of the scale and the lack of correlations of item 2 do not invalidate the SBST and we do not warrant a revision of this instrument. Since the SBST includes predictors of poor outcomes, its items might follow a formative model, i.e., a measurement model where the content of the construct is defined by its indicators, rather than a reflective one, i.e., a measurement model where the construct is assumed to be a latent factor influencing the individual's response to the items. Therefore, low correlations between the items could be expected (Bollen and Diamantopoulos, 2017). Since comorbid pain is an important risk factor for disability, item 2 should not be removed in order to avoid a loss of predictive power of the questionnaire. Therefore, we warrant further studies on the predictive ability of the SBST and of its items, which could provide a more sound basis to proceed with a revision of the scale.

Overall, the Italian version of the SBST had adequate psychometric properties. The test-retest reliability of the total score and of the subscale scores of the SBST was good, suggesting the questionnaire is a reliable method of assessing prognostic factors for disability outcomes in both obese and non-obese patients with ABP. This is consistent with other studies finding that the SBST has moderate to excellent test-retest reliability (Luan et al., 2014; Raimundo et al., 2017; Robinson and Dagfinrud, 2017; Yilmaz Yelvar et al., 2019; Ami et al., 2020; Schmidt and Naidoo, 2020). The construct validity of the SBST was adequate. According to the pre-specified hypotheses, presence of moderate correlations between the SBST and comparison questionnaires assessing physical and psychological risk factors for pain-related disability can be used as an indicator that the questionnaire measures a similar construct, but without overlapping with them. The presence of a high correlation with the RMDQ has been reported elsewhere (Abedi et al., 2015; Ami et al., 2020) and can be explained by the fact that the presence of multiple risk factors is associated with limitations in daily activities and, therefore, more disability.

This study has several limitations. The main limitation concerns the differences between Sample 1 and Sample 2, that included patients with different clinical manifestations and disability levels. This issue was partly by performing separate reliability and validity analyses on the two samples. In addition, the fact that the participants of this study were enrolled from a tertiary care hospital and a clinical osteopathic center limits the generalizability of the results to other clinical settings. Finally, the test-retest analysis could have been influenced by the fact that patients received treatments between the first and the second administration of the SBST. In conclusion, the SBST is a reliable and valid tool that can be used to measure physical and psychological risk factors for poor treatment outcomes in patients with low back pain with or without comorbid obesity.

## Data Availability Statement

The raw data supporting the conclusions of this article will be made available by the authors, without undue reservation.

## Ethics Statement

The studies involving human participants were reviewed and approved by Ethics Review Committee of the Accademia Italiana di Medicina Osteopatica. The patients/participants provided their written informed consent to participate in this study.

## Author Contributions

AM, AA, GC, GV, and EG made substantial contribution to the conception and design of the work and were responsible for drafting the manuscript. All authors critically revised it for important intellectual content, gave final approval to the finished manuscript, and agree to be accountable for all aspects of the work.

## Conflict of Interest

The authors declare that the research was conducted in the absence of any commercial or financial relationships that could be construed as a potential conflict of interest.

## Publisher's Note

All claims expressed in this article are solely those of the authors and do not necessarily represent those of their affiliated organizations, or those of the publisher, the editors and the reviewers. Any product that may be evaluated in this article, or claim that may be made by its manufacturer, is not guaranteed or endorsed by the publisher.
